# MicroRNA-451 Modulates Autophagy-Related Signaling with Relevance to Renal Fibrosis in an Accelerated Mouse Model of Diabetic Kidney Disease

**DOI:** 10.3390/cimb48020223

**Published:** 2026-02-19

**Authors:** Chidera Obiwuma, Baiyee-Ndang Agbor-Baiyee, Sadaf Ghaderzadeh, Neal Mohit, Kanwal K. Gambhir, Bradley Bobga, Maurice B. Fluitt

**Affiliations:** 1 Laboratory of Epigenetic and Metabolic Research, Division of Endocrinology, Department of Medicine, Howard University College of Medicine, Washington, DC 20059, USA; 2 Division of Endocrinology and Metabolism, Department of Medicine, Georgetown University, Washington, DC 20007, USA; 3 Laboratory of Endocrine and Metabolic Research, Division of Endocrinology, Department of Medicine, Howard University College of Medicine, Washington, DC 20059, USA

**Keywords:** diabetic kidney disease, microRNAs, autophagy, BTBR mice, locked nucleic acid, diabetic nephropathy

## Abstract

Background: Diabetic nephropathy is characterized by metabolic dysregulation, renal fibrosis, and impaired autophagy. MicroRNA-451 (miR-451) has been implicated in metabolic and stress-response pathways, but its role in diabetic kidney disease remains unclear. This study examined the effects of systemic miR-451 overexpression on renal injury and autophagy in BTBR *ob/ob* mice. Methods: Wild-type (WT) and BTBR *ob/ob* (OB) mice were treated with miR-451 mimics. Body weight, blood glucose, and urine albumin were assessed for three consecutive weeks. Renal miR-451 expression was measured by qRT-PCR, while protein levels of YWHAZ, mTOR, and autophagy markers were analyzed by Western blotting. Renal fibrosis was evaluated using Masson’s trichrome staining. Results: OB mice exhibited increased body weight, hyperglycemia, and albuminuria compared with WT controls. miR-451 treatment resulted in robust renal overexpression of miR-451 in OB treated mice (8.4-fold, *p* = 0.039) but did not normalize metabolic parameters. miR-451 overexpression significantly reduced renal expression of YWHAZ and mTOR. Histological analysis revealed increased glomerular fibrosis in OB mice, which was significantly attenuated following miR-451 treatment in WT-treated and OB-treated mice. In addition, miR-451 treatment increased expression of autophagy-related proteins ATG101 and Beclin-1 and reduced the LC3-II/I ratio, indicating altered autophagic signaling. Conclusions: miR-451 overexpression attenuates renal fibrosis and modulates autophagy-associated pathways in diabetic kidney disease, independent of metabolic control, highlighting miR-451 as a potential therapeutic target for diabetic kidney disease.

## 1. Introduction

Diabetes mellitus remains a global health challenge, with the International Diabetes Federation reporting that approximately 589 million adults living with the condition in 2024—a number projected to rise to 853 million by 2025 [1]. Among its many complications, DKD stands out as a major cause of morbidity and mortality. The kidneys are particularly vulnerable to microvascular damage in diabetes, and nearly 40% of diabetic patients eventually develop DKD [2,3]. This condition is a leading contributor to end-stage renal disease (ESRD) and is closely linked to increased cardiovascular mortality [4,5].

DKD is characterized by a range of pathological features, including glomerular basement membrane thickening, mesangial expansion, podocyte loss, and tubulointerstitial fibrosis. These structural changes contribute to progressive decline in renal function and are driven by a complex interplay of pathogenic mechanisms. Key contributors include dysregulation of the renin-angiotensin-aldosterone system, aberrant activity of growth factors and cytokines, and disruptions in metabolic pathways such as autophagy [6,7,8,9,10,11,12]. Although early interventions—primarily targeting blood pressure and glycemic control—can slow the progression of DKD, they are insufficient to fully prevent its advancement to ESRD. This underscores the urgent need to identify novel biomarkers and therapeutic targets by deepening our understanding of the molecular drivers of DKD.

Emerging evidence highlights the pivotal role of epigenetic regulators, particularly microRNAs (miRNAs), in DKD pathogenesis. MiRNAs are small, non-coding RNAs (18–22 nucleotides) that modulate gene expression post-transcriptionally through mRNA degradation or translational repression. In DKD, several miRNAs are differentially expressed and have been implicated in key pathological processes including fibrosis, inflammation, and metabolic dysregulation [13,14,15,16,17,18,19,20,21,22,23,24,25,26,27,28,29]. As such, miRNAs offer promising insights into disease mechanisms and may serve as both biomarkers and therapeutic targets in DKD. MiR-451 has emerged as a potential marker and mediator in DKD development and progression [30,31,32,33]. Its potential to modulate pathways involved in inflammation, fibrosis, and cellular stress response makes it a candidate of significant interest for future DKD research and therapeutic development. MiR-451 is a highly conserved miRNA known for its roles erythroid cell maturation and metabolic regulation [33,34]. Our previous work suggests that miR-451 may exert renoprotective effects by enhancing autophagy in diabetic settings [32].

Autophagy is a critical cellular process that maintains renal homeostasis by degrading and recycling damaged organelles and proteins, especially under stress conditions [35,36,37]. In DKD, impaired autophagic flux—particularly in podocytes and tubular epithelial cells—contributes to proteinuria, fibrosis, and cell death [38,39,40,41,42,43,44,45,46]. Although miRNAs are believed to influence autophagy, their specific roles in modulating this pathway in DKD remain incompletely understood.

In this study, we investigated the renoprotective effects of miR-451 in the BTBR *ob/ob* mouse model of DKD (an accelerated model of disease progression) using locked nucleic acid (LNA) miR-451 mimics. BTBR *ob/ob* mice, a type 2 diabetic animal model, lacking leptin due to a spontaneous mutation, develop severe type 2 diabetes, insulin resistance, and early-onset hyperglycemia (by 6-weeks in males and 8-weeks in females). These mice also exhibit renal lesions that closely mirror both early and advanced stages of human DKD [47,48]. Given the limited understanding of miR-451-mediated regulation of autophagy in diabetic kidney disease, this study aims to define the role of miR-451 in autophagy-related signaling using an attractive accelerated mouse model of diabetic kidney disease, thereby providing mechanistic insight into its contribution to renal injury.

## 2. Materials and Methods

### 2.1. Ethics Statement and Study Approval

All animal experiments were approved by the Howard University Institutional Animal Care and Use Committee (IACUC-MED-22-03, Approval Date: 31 October 2023) and were conducted in accordance with the National Institutes of Health Guide for the Care and Use of Laboratory Animals.

### 2.2. Animal Protocol

BTBR *ob/ob* and BTBR wild-type mice (strain #004824; The Jackson Laboratory, Bar Harbor, ME, USA), bred at Howard University, were studied between 12 and 15 weeks of age. Only male mice were included, as female BTBR ob/ob mice develop hyperglycemia at a later age and with less severity. Mice were randomly assigned to treatment groups at 11 weeks of age. Animals were housed at the Howard University Veterinary Service Facility (Howard University College of Medicine) under temperature-controlled conditions (20–22 °C) with a 12 h light/dark cycle, with a maximum of five mice per cage, and were provided standard chow and water ad libitum.

### 2.3. Study Design

At 11 weeks of age, BTBR *ob/ob* mice and WT mice were randomly divided into 4 groups: BTBR WT (WT) (*N* = 5), BTBR *ob/ob* (OB) (*N* = 4), BTBR WT treated with miR-451 mimic (WT+miR) (*N* = 8), and BTBR *ob/ob* treated with miR-451 (OB+miR) (*N* = 7). Inclusion criteria were defined to ensure clear phenotypic separation between normoglycemic controls and diabetic mice. WT mice were included if they exhibited a body weight between 20 and 30 g and fasting blood glucose levels between 100 and 150 mg/dL. BTBR ob/ob mice were included if they exhibited obesity, defined as a body weight between 40 and 50 g, and established hyperglycemia, defined as fasting blood glucose levels between 300 and 450 mg/dL. Fasting blood glucose measurements were obtained on two independent occasions prior to randomization. Mice were excluded if they fell outside these predefined ranges or exhibited signs of illness or distress. Animals meeting all inclusion criteria were randomly assigned to treatment or control groups for subsequent analyses. At 12 weeks of age, mice received an intraperitoneal injection of LNA-miR-451a mimic (2 mg/kg) once a week for 3-consecutive weeks (12, 13, and 14 weeks). Lyophilized LNA mimic probe (product name: mmu-miR-451a; mature miRNA sequence 5′-AAACCGUUACCAUUACUGAGUU-3′) was purchased from Qiagen (Qiagen, Germantown, MD, USA; cat. No. MSY0001632) and resuspended in 1 mL of sterile RNase-free water to a final concentration of 20 nmol. Spot urine was collected once a week at 12, 13, and 14 weeks, according to the Spot Urine Collection Protocol provided by the Mouse Metabolic Phenotyping Centers [49]. Body weight was measured once a week. Random blood glucose was measured from tail vein using a glucometer once a week. Mice were euthanized at the end of week 14, and kidneys and blood collected via exsanguination under anesthesia using isoflurane (at concentrations of 2% to 3%, at a flow rate of 0.4–0.8 L/min). Humane endpoints were predefined and included excessive weight loss (exceeding 20% of baseline), severe lethargy, inability to eat or drink, signs of respiratory distress, or any condition causing undue suffering (illness, injury, infection, or any condition judged by veterinary staff to cause undue suffering), at which point animals were humanely euthanized in accordance with IACUC and AVMA guidelines. No animals reached or met the predefined human endpoint criteria during the study. Group allocation was known only to the principal investigator during allocation and conduct of the experiment, while investigators performing outcome assessments and data analysis were blinded to group assignments.

### 2.4. Blood and Urine Analysis

Blood was collected via cardiac puncture, serum was separated by centrifugation and stored at −80 until further use. Spot urine samples were assessed for concentration using the Mouse Albumin ELISA Kit (Crystal Chem; Elk Grove Village, IL, USA; cat. No. 80630); according to the manufacturer’s instructions.

### 2.5. Kidney Tissue Histology

At euthanasia, mice were perfused with 1× phosphate-buffered saline (PBS), and kidneys were harvested. The left kidney was processed for RNA and protein expression analyses, whereas the right kidney was fixed with 4% paraformaldehyde for histological evaluation. Fixed kidneys were sectioned and stained with Masson’s trichrome at the Histopathology and Tissue Shared Resources Center (Georgetown University). Histological samples were visualized on a Nikon Eclipse Ni with Nikon camera. Color images were captured using a Nikon Digital Sight DS-Fi3 color camera. Images were taken with a 60× Plan Apo ojective lens. Acquisition was performed using NIS-Elements Basic Research software, version 5.30 (Nikon Instruments Inc., Melville, NY, USA). Quantitative analysis of Masson’s trichrome—stained sections was performed using ImageJ software (version 1.5r) according to the protocol outlined by Chen et al. (2017), with investigators blinded to experimental groups during image acquisition and analysis [50].

### 2.6. Quantitative Real-Time PCR

Total RNA was extracted from whole kidneys using miRNeasy tissue/cells advanced kit (Qiagen, Germantown, MD, USA; Cat No. 217684). Reverse transcription was performed using miRCURY LNA RT kit (Qiagen, Germantown, MD, USA; Cat No. 339340). Quantitative RT-PCR was performed in triplicate using an miRCURY LNA SYBR Green PCR kit (Qiagen, Germantown, MD, USA; Cat No. 339345) and miRCURY LNA miRNA PCR Primer Assay for miR-451a (Qiagen, Germantown, MD, USA; Cat No. 339306). Amplification followed by a melting curve step to confirm the specificity of amplification was carried out in a 96-well plate using the Applied Biosystems OneStep-Plus (Thermo Fisher Scientific, Waltham, MA, USA. All procedures were performed as outlined by the manufacturer. Data were normalized using GAPDH (Qiagen, Germantown, MD, USA; Cat No. 330001) as an endogenous control. Changes in miRNA gene expression were calculated using the ΔΔCt method [51].

### 2.7. Western Blot Analysis

Western blot analysis was performed on whole kidney homogenate from the left kidney and homogenized as previously described [52]. Briefly, cortex homogenates were solubilized in Laemmli sample buffer, and 10–20 µg of protein were loaded in precast 10% or 15%-gels, where appropriate (Bio-Rad Mini Protean TGX Gels, Bio-Rad Laboratories, Inc., Hercules, CA, USA). Separated proteins were transferred to nitrocellulose membranes (Bio-Rad Laboratories, Inc., Hercules, CA, USA; Cat No: 1704158) and blocked with 5% nonfat milk for 1 h. Membranes were incubated with respective primary antibodies, including YWHAZ, 14-3-3ς (Cell Signaling Technology, Danvers, MA, USA; rabbit monoclonal, D7H5,), mTOR (Cell Signaling Technology, Danvers, MA, USA; rabbit monoclonal, 2983), Beclin-1 (Abcam, Waltham, MA, USA; rabbit monoclonal, ab207612), ATG-101 (Cell Signaling Technology, Danvers, MA, USA; rabbit monoclonal, 13492), and LC3B (Abcam; rabbit polyclonal, ab48394). Protein loading was normalized by Ponceau-stained membranes before probing with primary antibodies. Blots were visualized using chemiluminescence via the Invitrogen iBright FL1000 Imager (Thermo Fisher Scientific, Waltham, MA, USA). Fluorescent blots were captured using the appropriate channels via Smart Exposure technology to minimize overexposure. Protein bands were quantified by densitometry using ImageJ software (National Institutes of Health, Bethesda, MD, USA), with band intensities normalized to Ponceau-stain [53].

### 2.8. Statistical Analysis

Data distribution was assessed for normality using the Shapiro–Wilk test. For non-normally distributed data, results are presented as median with interquartile range (IQR; minimum–maximum), and statistical comparisons were performed using the Kruskal–Wallis test followed by pairwise comparisons. For normally distributed data, the resulting data are presented as mean ± SE. Statistical analyses were performed using repeated measures ANOVA, or one-way ANOVA, as appropriate, with Bonferroni correction applied for multiple comparisons. All analyses were conducted using IBM SPSS Statistics for Mac, Version 30.0.0.0 (171) (IBM Corp., Armonk, NY, USA) and a *p* < 0.05 was considered statistically significant.

## 3. Results

### 3.1. Metabolic and Renal Parameters

**Figure 1:**
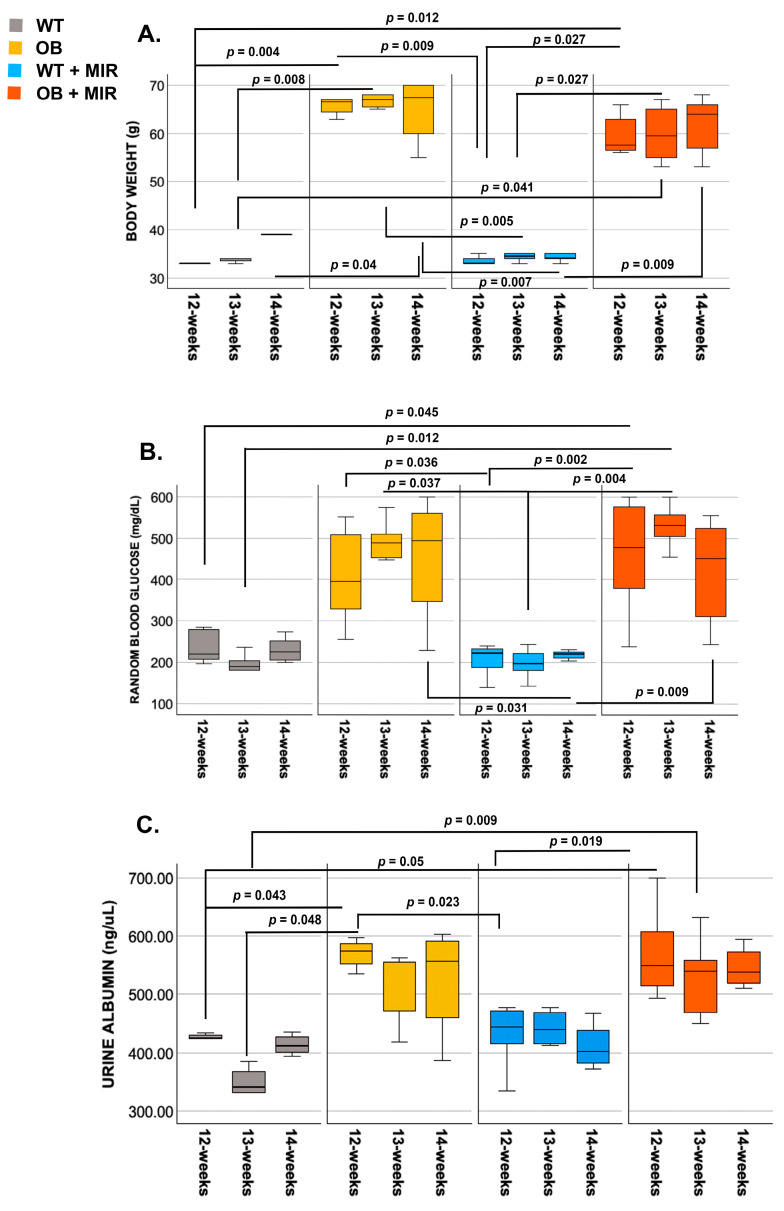
Box-and-whisker plots showing values for (**A**) body weight, (**B**) blood glucose, and (**C**) urine albumin in WT, OB, WT+miR, and OB+miR mice across time points. Data are presented as median with interquartile range (minimum–maximum). Statistical analysis was performed using the independent-samples Kruskal–Wallis test followed by pairwise comparisons. *p*-values were adjusted using the Bonferroni correction for multiple testing. Statistical significance was defined as *p* < 0.05.

Across the study period, body weight, blood glucose, and urine albumin levels exhibited clear group-dependent differences (Figure 1). At 12 weeks, median body weight was significantly higher in OB (1.94-fold; *p* = 0.012) and OB+miR mice (1.12-fold; *p* = 0.027) compared with WT and WT+miR controls. This difference persisted at 13 weeks, with median body weight remaining elevated in both OB (1.94-fold) and OB+miR mice (1.73-fold) relative to WT groups. By 14 weeks, OB (67.2 g; IQR, 57.7–69.9) and OB+miR mice (63.6 g; IQR, 56.5–67.2) continued to exhibit increased median body weight; however, OB+miR mice no longer differed significantly from WT animals at this time point (Figure 1A).

A similar trend was observed for blood glucose. At 12 weeks, median blood glucose was 1.65-fold higher in OB mice (*p* = 0.036) and 2.42-fold higher in OB+miR mice (*p* = 0.002) compared with WT and WT+miR controls. These differences were maintained at 13 weeks, with OB and OB+miR mice exhibiting 2.5-fold (*p* = 0.037) and 2.91-fold (*p* = 0.004) higher glucose levels, respectively. At 14 weeks, blood glucose remained elevated in OB (2.2-fold; *p* = 0.031) and OB+miR mice (2.0-fold; *p* = 0.009) relative to WT+miR animals, while untreated WT mice did not differ significantly from OB mice (Figure 1B).

Urine albumin levels were also increased in obese mice. At 12 weeks, OB and OB+miR mice exhibited 1.3-fold (*p* = 0.04) and 1.4-fold higher median urine albumin levels, respectively, compared with WT and WT+miR controls. At 13 weeks, urine albumin remained elevated in OB (1.5-fold; *p* = 0.04) and OB+miR mice (1.6-fold; *p* = 0.009) relative to WT mice, although values were not significantly different from WT+miR animals. By 14 weeks, urine albumin levels no longer differed significantly among groups (Figure 1C).

### 3.2. Renal miR-451 Expression

**Figure 2:**
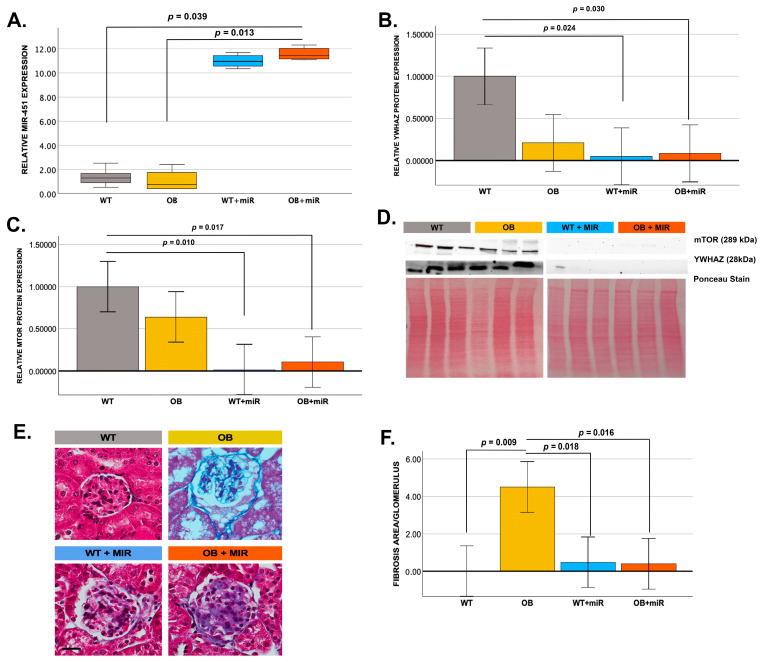
Treatment with miR-451 mimic increases renal miR-451 expression and improves renal fibrosis in BTBR mice. (**A**) miR-451 expression in whole-kidney homogenates (normalized to GAPDH) from untreated WT (*N* = 5) and OB (*N* = 5) mice, and miR-451–treated WT+miR (*N* = 4) and OB+miR (*N* = 6) mice. (**B**–**D**) Western blot analysis and relative band-density quantification (normalized to Ponceau stain) for the predicted miR-451 target YWHAZ (**D**) and for mTOR (**E**) in WT, OB, WT+miR, and OB+miR mice (*N* = 3/group). (**E**) Representative Masson’s Trichrome staining and (**F**) quantitative analysis of fibrosis (*N* = 3/group; 50 µm scale). In panel (**A**), data are presented as median with interquartile range (minimum–maximum) and were analyzed using the independent-samples Kruskal–Wallis test followed by pairwise comparisons where appropriate. For panels (**C**–**E**), data are presented as mean ± SE and analyzed using one-way ANOVA, with *p*-values adjusted using the Bonferroni correction for multiple comparisons. Statistical significance was defined as *p* < 0.05.

To assess renal miR-451 expression, qRT-PCR analysis was performed on kidney tissue from WT, OB, WT+miR-451, and OB+miR-451 mice. Systemic administration of miR-451 mimics resulted in an 8.4-fold increase in renal miR-451 expression in OB+miR mice compared with both untreated WT and OB animals, whereas WT+miR mice did not exhibit increased expression relative to controls (Figure 2A).

### 3.3. Validation of miR-451 Target Suppression

Consistent with miR-451 overexpression, expression of the predicted target YWHAZ was significantly reduced in WT+miR mice (*p* = 0.024) and OB+miR mice (*p* = 0.030) compared with WT controls (Figure 2B). In parallel, renal mTOR protein expression was significantly reduced in both WT+miR mice (*p* = 0.010) and OB+miR mice (*p* = 0.017) relative to WT controls (Figure 2C).

### 3.4. Effect of miR-451 on Renal Fibrosis

Histological analysis demonstrated minimal glomerular fibrosis in WT control mice, whereas fibrosis scores were significantly increased in OB mice (4.51 ± 0.96; *p* = 0.009). In contrast, miR-451 treatment significantly reduced glomerular fibrosis in both WT+miR (0.48 ± 0.96; *p* = 0.018) and OB+miR mice (0.40 ± 0.96; *p* = 0.016) compared with untreated OB mice (Figure 2E,F).

### 3.5. miR-451 Modulates Renal Autophagy Markers

**Figure 3:**
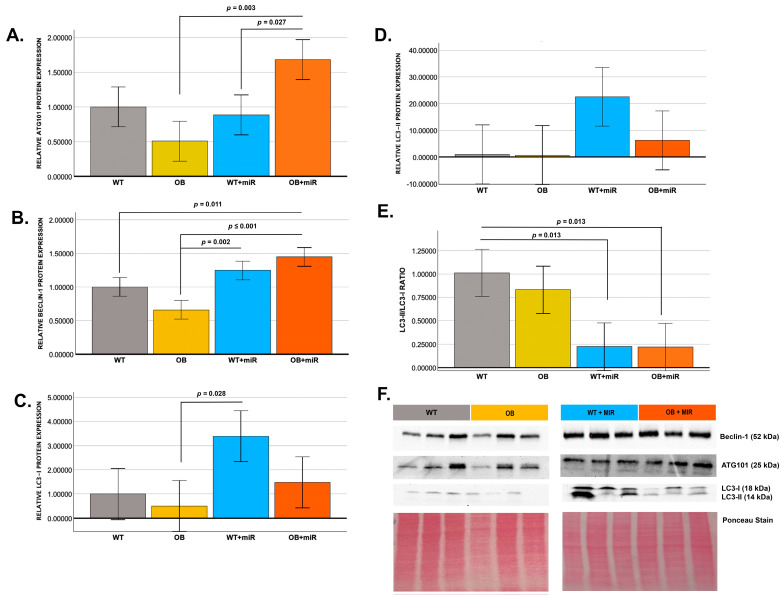
miR-451 treatment triggers early stages of autophagy in kidneys of diabetic BTBR mice. Western blot analysis of (**A**) ATG-101, (**B**) Beclin-1, (**C**) LC3-I, (**D**) LC3-II, (**E**) LC3-II/LC3-I ratio, and (**F**) relative band-density summaries (normalized to Ponceau stain) for Beclin-1, ATG-101, LC3-I, and LC3-II (*N* = 3/group) for WT, OB, WT+miR, and OB+miR mice. Data are presented as mean ± SE and analyzed using one-way ANOVA, with *p*-values adjusted using the Bonferroni correction for multiple comparisons. Statistical significance was defined as *p* < 0.05.

To evaluate autophagy-related signaling, protein expression of ATG101, Beclin-1, and LC3 was assessed by Western blotting. miR-451 treatment significantly increased ATG-101 expression in OB+miR mice compared with untreated OB mice and WT+miR animals (*p* = 0.003 and *p* = 0.027, respectively) (Figure 3A). Beclin-1 expression was also significantly increased in WT+miR mice relative to untreated OB mice (*p* = 0.002) and in OB+miR mice compared with both untreated WT and OB groups (*p* < 0.001) (Figure 3B). Analysis of LC3 isoforms demonstrated increased LC3-I expression in WT+miR mice compared with untreated OB animals (*p* = 0.028) (Figure 3C), while LC3-II levels did not differ among groups (Figure 3D). However, the LC3-II/I ratio was significantly reduced in both WT+miR and OB+miR mice relative to WT controls (Figure 3E).

## 4. Discussion

Autophagy is a genetically regulated and evolutionarily conserved process essential for maintaining cellular homeostasis through the degradation of damaged organelles and proteins. In the kidney, impaired autophagic signaling has been linked to the onset and progression of diabetic kidney disease [54,55]; however, the specific role of miRNAs, particularly miR-451, remains incompletely understood. Prior studies suggest that miR-451 may exert protective effects under diabetic conditions by suppressing cellular proliferation and enhancing autophagic activity in the kidney [32]. Building on this evidence, we investigated the renoprotective effects of miR-451 in DKD using BTBR *ob/ob* mice, a robust and attractive model of DKD.

### 4.1. miR-451 Modulation of Fibrosis Associated Signaling in Diabetic Kidney Disease

Dysregulation of miR-451 has been implicated in several pathological conditions, including DKD, where its expression is often reduced [21,30,31,32,55,56,57,58]. In the current study, we found that administering a single low dose of miR-451 mimic once a week for three consecutive weeks significantly increased renal miR-451 expression—by approximately 8-fold in OB+miR mice. Restoring miR-451 expression was associated with a significant reduction in glomerular collagen in WT- and OB+miR mice, in comparison to untreated OB mice, suggesting improved fibrosis. Xu et al. recently reported on the effects of miR-451 on renal fibrosis in Sprague Dawley rats. They reported that upregulation of miR-451 improves renal fibrosis by regulating downstream fibrosis-related factors, achieved through treatment with a Chinese medicine Zhenwu Decocotion [56]. These findings further highlight the renoprotective effects of miR-451 in the diabetic kidney.

Mechanistically, miR-451 overexpression was accompanied by reduced expression of YWHAZ (14-3-3ζ), a predicted target of miR-451, in both WT+miR and OB+miR mice. YWHAZ is a multifunctional scaffold protein involved in protein trafficking, cell proliferation, apoptosis, and autophagy [38]. In DKD, YWHAZ interacts with components of the mTOR pathway, contributing to autophagy suppression and fibrosis [21,32,59]. Zhang et al. (2012) reported that miR-451 inhibits YWHAZ and downstream p38 MAPK signaling, reducing mesangial hypertrophy in early diabetic nephropathy [21].

Consistent with these observations, miR-451 mimic treatment in our study reduced renal mTOR expression, in contrast to reports showing increased mTOR activity following miR-451 inhibition in insulin-resistant models. These findings further support the role of the miR-451/YWHAZ/mTOR axis in regulating autophagy in DKD. However, additional studies are needed to fully clarify its impact on autophagy response and fibrosis resolution in the BTBR *ob/ob* model of DKD.

### 4.2. MiR-451 Modulates Early Autophagy Signaling in BTBR ob/ob Mice

To further assess the effects of miR-451 mimic treatment on DKD progression in BTBR WT and BTBR *ob/ob* mice, we evaluated protein expression of key autophagy markers, including Beclin-1, ATG101, and LC3 isoforms (LC3-I and LC3-II). Beclin-1 is a core component of the class III PI3K complex and plays a pivotal role in autophagy initiation by promoting phagophore nucleation and coordinating vesicle trafficking [44]. Western blot analysis revealed that untreated OB mice exhibited significantly reduced Beclin-1 expression compared to WT+miR and OB+miR mice, in agreement with previous findings highlighting impaired autophagy signaling in diabetic kidneys, and [54,60,61,62] suggests that miR-451 enhances the early stages of autophagy initiation under both normal and diabetic conditions. This is supported by prior evidence suggesting that miR-451 regulates autophagy-related pathways, including AMPK and PI3K signaling, which are critical for Beclin-1 activation [26]. AMPK promotes autophagy by inhibiting mTORC1 and directly phosphorylating key autophagy-related proteins, including ULK1 and Beclin-1. During early autophagy, Beclin1-regulated autophagy protein 1 (AMBRA1) facilitates ULK1 activation through K63-linked polyubiquitination, enhancing its kinase activity and promoting Beclin-1-dependent autophagic signaling [62,63,64]. Given this pathway, miR-451 may regulate autophagy in the current model by modulating upstream signals that influences Beclin-1 activation in our model.

Autophagy initiation is tightly regulated by the ULK1 complex, which includes ULK1, FIP200, ATG13, and ATG101 [65,66,67,68,69]. Among these, ATG101 functions as a scaffold and stabilizer, ensuring the structural integrity of the complex and facilitating its recruitment to the phagophore assembly site. It supports ULK1 kinase activity, which is essential for phosphorylating downstream autophagy effectors such as Beclin-1. Notably, AMPK activation—a known target of miR-451—can stimulate autophagy by inhibiting mTORC1 and directly phosphorylating ULK1 and Beclin-1 [70]. In the present study, miR-451 treatment significantly increased ATG101 protein expression in the OB+miR group relative to untreated BTBR WT and untreated BTBR *ob/ob* mice. Given that miR-451 has been shown to modulate AMPK activity [64,70,71], it may indirectly regulate autophagy initiation by influencing the ULK1 complex through ATG101 and promoting Beclin-1–dependent signaling under diabetic conditions.

LC3 proteins are central regulators and markers of autophagy [72]. LC3 is initially synthesized as a cytosolic precursor, LC3-I, which is cleaved by ATG4 and subsequently lipidated via a ubiquitin-like conjugation system involving ATG7 and ATG3 [72,73,74]. This lipidation converts LC3-I into LC3-II, the membrane-bound form that integrates into autophagosomes and facilitates their elongation, maturation, and cargo recruitment. LC3-II is a reliable marker of autophagosome formation [72,73,74]. Under our current low dose treatment scheme, untreated OB mice exhibited a modest reduction in LC3-I protein levels, consistent with impaired autophagy initiation under diabetic conditions. Conversely, miR-451 treatment significantly enhanced LC3-I expression in WT+miR mice compared to untreated WT and OB mice. However, we did not detect significant changes in LC3-I in the OB+miR group. This suggests that effects of miR-451 on autophagy response may be more complex in diabetic and obese mice. Additionally, we did not detect differences in LC3-II expression in any of the four groups. The current findings imply partial autophagy initiation or impaired modulation of autophagy-related signaling.

The discrepancy observed between LC3-I and LC3-II levels may reflect limitations in the current assessment of autophagy rather than definitive impairment of autophagic flux. One potential interpretive framework, described in prior studies, is the concept of “autophagic stagnation,” a pathological state in which autophagy initiation occurs but downstream processes fail to progress efficiently under conditions of sustained metabolic stress [74,75,76,77,78,79]. This phenomenon has been reported in models of obesity and type 2 diabetes, where excessive lipid accumulation, organelle damage, and proteotoxic stress may overwhelm autophagic capacity. BTBR *ob/ob* mice represent a model of severe obesity and insulin resistance accompanied by hypertriglyceridemia and systemic lipid overload, conditions that have been associated with disrupted autophagy in renal cells [75,76,77,78,79]. However, because autophagic flux was not directly measured in the present study, we cannot conclude that autophagic stagnation occurred in this model. Rather, our findings indicate modulation of early autophagy-related signaling without corresponding changes in LC3 lipidation, underscoring the need for more comprehensive assessment of downstream autophagic processes. Accordingly, future studies incorporating direct measurements of autophagic flux—such as LC3-II turnover assays, p62/SQSTM1 degradation, and lysosomal inhibition—will be necessary to determine whether impaired flux contributes to renal injury in this model and whether enhanced or prolonged miR-451 modulation can overcome metabolic constraints imposed by severe obesity.

### 4.3. Study Limitations

This study has several limitations. First, the experimental design used a relatively small sample size and included only male BTBR *ob/ob* mice, which limits generalizability and precludes assessment of sex-specific responses. Second, the dosing regimen consisted of a low dose of LNA–miR-451 mimic administered once weekly for three consecutive weeks. While this was sufficient to modulate select signaling pathways in the current model, the short duration and limited dose range prevent full characterization of the dose–response relationship or long-term therapeutic potential. Additionally, the absence of an LNA-scramble control restricts precise evaluation of off-target or sequence-independent effects. Moreover, measurements were performed using whole-kidney homogenates, which obscure cell-specific responses in distinct renal compartments known to differentially regulate autophagy and fibrosis [80,81,82,83]. In addition, although changes in early autophagy markers were observed, autophagic flux was not directly assessed using standard assays such as LC3-II turnover or p62/SQSTM1 degradation, limiting interpretation of whether miR-451 affects autophagosome maturation. Finally, functional measures of renal injury, including BUN, creatinine, and eGFR, were not included, and systemic administration of miR-451 mimic precludes differentiation between direct renal versus extrarenal effects.

### 4.4. Future Directions

Future studies should expand these findings by incorporating both male and female BTBR mice and employing larger cohorts to enhance statistical robustness. In addition, dose-escalation and extended-duration treatment studies will be essential to determine the optimal delivery strategy and to evaluate long-term renal protection during disease progression. Moreover, inclusion of an LNA-scramble control and tissue-specific delivery approaches will help to define target specificity and minimize systemic confounding off target effects. To further understand cell-type-specific effects, cell-specific analyses, such as laser-capture microdissection, single-cell sequencing, or renal cell lineage reporters, are needed to identify the precise renal cell populations regulated by miR-451. Importantly, comprehensive assessment of autophagic flux using LC3-II turnover, p62 degradation, and lysosomal inhibition will clarify the stage(s) of autophagy affected by miR-451. Future work should also incorporate functional measures of kidney injury to further evaluate whether miR-451 improves renal function. Ultimately, extending these findings to human DKD tissues or patient-derived exosomes will also provide critical insight and foundational support to establish translational relevance and determine whether miR-451 can serve as a therapeutic target in human diabetic kidney disease.

## 5. Conclusions

Collectively, this study demonstrates that miR-451 modulates autophagy-associated signaling in the diabetic kidney, as evidence by increased expression of key initiation regulators, including Beclin-1 and ATG101, in the BTBR *ob/ob* mouse model. These findings are consistent with prior reports implicating miR-451 in the regulation of autophagy-related pathways involving AMPK and mTOR signaling [32,66,84,85,86,87,88]. Notably, alterations in early autophagy markers were not accompanied by corresponding changes in LC3 lipidation, suggesting that miR-451 may preferentially modulate early stages of autophagy in this model. The impact of miR-451 on downstream autophagic processes, including autophagosome maturation and flux, therefore remains to be determined [89,90,91].

Despite these limitations, the present findings provide mechanistic insight into the role of miR-451 in autophagy-related signaling within the diabetic kidney and support its involvement in pathways relevant to renal injury. Together, these data suggest that miR-451 may contribute to the regulation of early autophagic responses in diabetic kidney disease, warranting further investigation into its biological significance.

## Data Availability

The original contributions presented in this study are included in the article. Further inquiries can be directed to the corresponding author.

## Abbreviations

The following abbreviations are used in this manuscript:

DKD	Diabetic Kidney Disease
miRNA	MicroRNA
AMPK	AMP-activated protein kinase
WT	BTBR Wild Type mice
OB	BTBR *ob/ob* mice
WT+miR	BTBR WT miR-451 treated mice
OB+miR	BTBR *ob/ob* miR-451 treated mice
